# Carbon Cycling in Wetlands Under the Shadow of Microplastics: Challenges and Prospects

**DOI:** 10.3390/toxics13030143

**Published:** 2025-02-20

**Authors:** Linan Liu, Yizi Hua, Jingmin Sun, Shakeel Ahmad, Xin He, Yuguo Zhuo, Jingchun Tang

**Affiliations:** 1 Hebei Provincial Key Laboratory of Agroecological Safety, Hebei Engineering Research Center for Ecological Restoration of Seaward Rivers and Coastal Waters, Hebei University of Environmental Engineering, Qinhuangdao 066102, China; liulinan@nankai.edu.cn (L.L.); sjmustb@163.com (J.S.); hex80@163.com (X.H.); zhuoyuguo@hebuee.edu.cn (Y.Z.); 2 MOE Key Laboratory of Pollution Processes and Environmental Criteria, Tianjin Engineering Center of Environmental Diagnosis and Contamination Remediation, College of Environmental Science and Engineering, Nankai University, Tianjin 300350, China; hua_yizi@163.com; 3 Faculty of Environmental Science and Engineering, Kunming University of Science and Technology, Kunming 650500, China; shakeel@kust.edu.cn

**Keywords:** dissolved organic matter, microbial communities, carbon sequestration, greenhouse gas emissions, ecological toxicology

## Abstract

Wetlands are one of the most crucial ecosystems for regulating carbon sequestration and mitigating global climate change. However, the disturbance to carbon dynamics caused by microplastics (MPs) in wetlands cannot be overlooked. This review explores the impacts of MPs on the carbon cycles within wetland ecosystems, focusing on the underlying physicochemical and microbial mechanisms. The accumulation of MPs in wetland sediments can severely destabilize plant root functions, disrupting water, nutrient, and oxygen transport, thereby reducing plant biomass development. Although MPs may temporarily enhance carbon storage, they ultimately accelerate the mineralization of organic carbon, leading to increased atmospheric carbon dioxide emissions and undermining long-term carbon sequestration. A critical aspect of this process involves shifts in microbial community structures driven by selective microbial colonization on MPs, which affect organic carbon decomposition and methane production, thus posing a threat to greenhouse gas emissions. Notably, dissolved organic matter derived from biodegradable MPs can promote the photoaging of coexisting MPs, enhancing the release of harmful substances from aged MPs and further impacting microbial-associated carbon dynamics due to disrupted metabolic activity. Therefore, it is imperative to deepen our understanding of the adverse effects and mechanisms of MPs on wetland health and carbon cycles. Future strategies should incorporate microbial regulation and ecological engineering techniques to develop effective methodologies aimed at maintaining the sustainable carbon sequestration capacity of wetlands affected by MP contamination.

## 1. Introduction

Wetlands are among the most critical ecosystems for regulating global carbon cycling, acting as significant sinks for carbon dioxide (CO_2_) and sources of methane (CH_4_) emissions [1,2]. Increasingly, researchers are focusing on elucidating the processes involved in wetland carbon cycling and identifying the controlling factors that influence these processes [3]. Both aquatic plant species and microbial communities, along with their associated habitats, play crucial roles in carbon storage, which can be affected by multiple physicochemical factors, including exogenous organic matter input, salinity changes, and interactions between iron minerals and organic matter [4,5]. Specifically, plant–soil–microorganism interactions have been shown to significantly affect carbon dynamics in tidal freshwater wetlands, with the abundance of rhizosphere iron plaques identified as a key factor influencing carbon decomposition rates [4]. Soil minerals are pivotal in buffering dissolved organic matter (DOM) retention in wetlands, particularly those rich in oxidizable iron content, supporting the “iron gate” mechanism for soil carbon protection [6]. This finding underscores the importance of mineral–DOM interactions, which may play a more decisive role than biological degradation in determining the fate of wetland carbon sequestration. Notably, microbial communities and their associated carbon/nitrogen metabolisms are considered primary drivers of organic matter degradation, which are highly sensitive to alterations in carbon sources, consequently influencing both the stabilization of soil organic carbon (SOC) and the emission rates of greenhouse gases [7,8]. However, the controlling factors of organic carbon dynamics in various types of wetlands and variations in habitats, and their subsequent impacts on carbon sequestration, remain largely unknown, which is crucial for assessing the long-term effects of wetland systems on climate warming.

Microplastics (MPs), one kind of prevalent permanent pollutant, along with their chemical additives and released substances (e.g., heavy metals, organic contaminants, or derived DOM), exert profound impacts on the physicochemical properties of soils/sediments and the structures of microbial communities [9,10]. Microplastics have proven to pose great challenges for global carbon cycling and the healthy development of animals, plants, and microorganisms, which are highly dependent on the types, sizes, and shapes of MPs and environmental changes, such as climate warming [11,12]. Specifically, the synergistic impact of ocean warming and MP pollution significantly decreased diatom growth and photosynthetic efficiency, thereby impeding carbon/nitrogen sequestration by allocating carbon into flexible carbohydrates [13]. Leachates from MPs are highly toxic, inhibiting the growth rate of microalgal cells and disrupting gene functions (e.g., translation, ribosomal structure, and biogenesis), potentially causing more severe toxicity than MPs themselves [14]. Additionally, the accumulation of MPs in paddy soils interferes with autotrophic microbial communities, leading to more random-dominated assemblages and a more fragile network compared to controls, ultimately reducing the carbon assimilation efficiency of autotrophic microorganisms and decreasing the carbon fixation activity of autotrophic bacteria [15]. These findings highlight that MPs can directly participate in carbon dynamics and indirectly regulate carbon metabolism by restructuring microbial community assembly patterns. Furthermore, DOM released from aged MPs (MPs-DOM) exhibits higher biological stability and mineralization rates, which may accelerate greenhouse gas emissions in aquatic environments. The impact induced by MPs-DOM is amplified in marine microbial activity, potentially up to two orders of magnitude higher than that triggered by pristine MPs, thus leading to non-negligible disruptions in the natural carbon cycle [16,17]. Notably, with the increasing replacement of traditional plastics by biodegradable plastics, which are easily fragmented into MP particles through physiochemical and biological processes in the environment, the toxic effects of biodegradable MPs on organisms within various ecosystems warrant greater attention compared to traditional MPs [18].

Although wetlands are recognized as pivotal ecosystems for MP enrichment and transportation, research on the specific impacts of MPs on wetland functions and carbon dynamics remains limited. Recent observations indicate that the accumulation of MPs within wetlands significantly elevates sediment carbon content, altering its physical and chemical properties while disrupting the original storage of organic carbon, particularly through interference with microbial community structure [19,20]. For instance, An et al. [21] employed ^13^C stable isotope and molecular techniques to demonstrate that both conventional and biodegradable MPs enhance CH_4_ emissions in estuarine and coastal wetlands, with biodegradable MPs exhibiting a more pronounced effect due to the accelerated activity of CH_4_-producing bacteria and inhibited CH_4_ oxidation. Similarly, MPs have been found to reduce bacterial diversity, alter soil enzyme activity associated with litter decomposition, and modify microbial community structure, resulting in decreased litter quality (high C:N ratio) and impacting key processes within the carbon and nitrogen cycles of wetlands [22]. Xie et al. [23] observed that polyamide and polyvinyl chloride MPs significantly impair the capacity of mangrove rhizosphere microbes to utilize preferred polymer carbon sources and hinder their activity in the biological transformation of nitrogen-containing carbon sources. This impairment reduces the efficiency of nutrient uptake by plants through changes in nutrient flow patterns [24]. Notably, the impact of MPs on vegetated wetlands varies based on shape and particle size; fibrous MPs are more readily absorbed or attached to plant roots [25], whereas smaller-sized particles can penetrate deeper into wetland environments [26]. Despite these findings, much of the existing research focuses predominantly on short-term experiments involving single-variable changes. Consequently, the ecological effects and physicochemical mechanisms of long-term MP exposure on wetland carbon cycling remain largely unexplored. Therefore, it is imperative to synthesize recent advancements in understanding MPs’ impacts within wetland ecosystems. Such synthesis will not only inform future investigations into the monitoring and management of MP pollution but also facilitate assessments of global wetlands’ carbon sequestration capacities under the influence of MPs.

Given the considerable potential for MPs to accumulate in wetland ecosystems and their detrimental effects on carbon sequestration, this review aims to explore the effects and underlying mechanisms of MPs’ disruption of wetland carbon dynamics based on the current research. Specifically, we investigate the accumulation of MPs across natural, artificial, and coastal wetlands, examining their diverse impacts on organic carbon, microbial community structure, carbon metabolism activity, and greenhouse gas emissions. This review seeks to achieve three objectives: (i) provide a comprehensive evaluation of MPs’ impact on wetland carbon sequestration and emissions; (ii) identify critical factors contributing to the reduction in wetlands’ carbon sequestration capacities induced by MPs; and (iii) propose targeted strategies for mitigating MP pollution in wetland ecosystems.

## 2. Distribution of Microplastics in Wetlands

Wetland ecosystems play a crucial role in environmental protection and climate regulation, particularly due to their significant carbon sink capacity, accounting for approximately 35% of the global terrestrial carbon storage [27]. Undisturbed peatlands and coastal blue carbon ecosystems, such as salt marshes, mangroves, and seagrass beds, serve as substantial carbon sinks; however, if degraded, they can become significant sources of greenhouse gases [28]. Research has demonstrated that climate change, exogenous carbon/nitrogen inputs, and interactions between soil conditions and plant growth can alter wetland carbon sequestration by influencing soil respiration, microbial biomass carbon, and microbial activity [29]. In recent decades, the continuous accumulation of emerging pollutant MPs in wetlands has led to significant alterations in carbon dynamics and disturbances in microbial community structures, posing potential threats to wetland health and sustainable development [30]. Zhao et al. [31] reported an average concentration of MPs in global wetland sediments as high as 2046.7 items kg^−1^. This phenomenon is attributed to the strong interception capability of wetlands, facilitated by the complex and extensive root systems of wetland plants, along with the large specific surface area and abundant organic matter present in sediments [32]. For instance, constructed wetlands can intercept over 90% of MP particles from wastewater [33]. Meanwhile, due to land-based human activities and marine pollution, estuarine and coastal areas have accumulated high concentrations of MPs (ranging from 1.38 to 7.95 million tons) [34,35].

Previous studies have shown that total organic carbon (TOC) and silt content in mangrove-dominated wetland sediments are critical factors promoting the deposition of MPs [36]. Small MP particles, including microfibers or plastic fragments primarily originating from synthetic textiles in wastewater discharges and larger plastic fragments, are particularly prevalent in mangrove sediments, indicating that nanoscale MPs represent one of the most concerning pollutants in these ecosystems [26]. Notably, the shape of MPs significantly influences their fate within vegetated wetlands. McIlwraith et al. [25] demonstrated that spherical MPs are more easily transported by water flow, eventually entering connected water bodies through wetlands, leading to cross-regional pollution. Conversely, fibrous MPs are more likely to adhere to plant roots or remain suspended in water, persisting within the wetlands. Different types of plant-dominated wetlands exhibit varying levels of MP interception and accumulation, with reed plants proving more effective than other wetland vegetation at trapping MPs and reducing their spread to downstream environments [37]. In artificial wetland ecosystems, biofilm attachment and physical filtration play crucial roles in retaining MPs, while the shape of MPs and the pore size of the matrix are closely linked to their transport within the substrate [38]. Nonetheless, the distribution, accumulation, and migration of MPs in wetlands are complex and multifaceted, with significant gaps remaining in our understanding of their dynamic behavior and influencing factors across different types of wetlands.

The presence of MPs in wetland ecosystems not only disturbs the physical properties of the wetlands but also affects soil enzyme activity and alters microbial community structure, thereby influencing the rate of organic matter degradation [22]. Due to their hydrophobic nature, MPs provide an important medium for hydrophobic organic chemicals, disrupting local microbiota that form critical symbiotic relationships, ultimately leading to negative impacts on soil quality and biodiversity [39]. Additionally, MPs interfere with the treatment performance of artificial wetlands, reducing the efficiency of pollutant removal, such as purifying nutrients like nitrogen and phosphorus, and can even impact the overall health of wetland ecosystems [33,40]. Therefore, exploring the specific mechanisms of long-term MP impacts on wetland ecosystems under natural conditions, as well as identifying effective management and restoration strategies, is paramount for protecting these vital ecological resources.

## 3. Toxic Impacts of Microplastics on the Wetland Rhizosphere

The detrimental impacts of MPs on wetland ecosystems are primarily manifested through alterations to the rhizosphere environment and interference with plant physiological functions and microbial communities, collectively resulting in various adverse outcomes for plant health [41]. Plants have the potential to absorb nano- or sub-micrometer-sized MPs through their root systems, which can be transported or accumulated within plant tissues, posing a substantial risk to human health if these MPs enter the food chain [42]. Changes in soil structure induced by MPs will disrupt soil moisture and nutrient availability, which further alter soil microbial communities and enzymes and undermine the stability of soil bacterial symbiotic networks that are crucial for the degradation of refractory carbon [43]. Soil aggregate structure, a property particularly susceptible to MP exposure, is altered by both traditional and degradable MPs, generally increasing the composition of microaggregates while reducing macroaggregates [43]. This shift directly influences soil organic carbon utilization, microbial community composition, and metabolic activity due to the marked variation in organic carbon availability among different aggregates [44]. Additionally, Lian et al. [45] illustrated that lettuce growth was significantly inhibited, with shoot length being reduced by more than 30% compared to controls, regardless of MP type and dosage. These findings were attributed to decreased soil bacterial diversity and disturbances to the plant antioxidant system, which were identified as critical factors affecting plant physiology, including photosynthesis processes and reactive oxygen species (ROS) production under MP exposure [45]. The reduction in soil bacterial diversity and the consequent impact on the plant’s antioxidant defense mechanisms highlight the profound influence of MPs on the fundamental biological processes of plants, emphasizing the need for further investigation into the long-term ecological consequences of MPs in wetland environments.

In wetland soils, MPs can significantly influence the transmission of water and oxygen by increasing soil compaction, reducing porosity, or obstructing plant cell pores. These changes limit root growth and impair a plant’s capacity to uptake nutrients and water [46]. Riaz et al. [47] investigated the distribution of MPs in mangrove ecosystems along the Karachi coastline in Pakistan, revealing higher concentrations of MPs in the rhizosphere compared to non-rhizosphere areas. This preferential accumulation within the rhizosphere poses a direct threat to plant health. Similarly, MP particles can inflict direct damage on plant roots, leading to cell wall rupture or increased membrane permeability, thereby disrupting normal physiological functions, such as photosynthesis and respiration, and ultimately affecting overall plant growth [48]. Adsorbed or released harmful chemicals, such as heavy metals or organic pollutants from MPs, negatively impact root growth or result in reproductive toxicity due to their transportation into plants [49]. The accumulation of MPs in higher plants and their rhizospheres can manifest as symptoms like stunted growth and leaf chlorosis, alongside shifts in the rhizosphere microbial community composition that reduce beneficial bacterial populations [50]. Such alterations may weaken the plant’s disease resistance and lower productivity.

Specific types of MPs, including polyethylene, polyvinyl chloride, and expanded polystyrene, affect the metabolic activity of dominant bacteria depending on the functional groups attached to polymer molecules, exhibiting selective effects on microbial communities within mangrove rhizospheres [51]. These microbial communities associated with MP biofilms further disrupt carbon or sulfur cycling, and their interactions with rhizosphere microbes can influence the development of mangrove plants. Additionally, MPs can serve as substrates for microbial attachment, promoting the proliferation of specific microorganisms and creating new ecological niches [52]. For instance, biofilms formed on MPs in polluted environments facilitate horizontal gene transfer between microbes, potentially accelerating the spread of antibiotic resistance genes and genes related to MP degradation [53]. The presence of MPs may also amplify the distribution of antibiotic-resistant genes by altering microbial community composition, including potential pathogens and antibiotic-resistant genes, posing an ecological risk concerning the spread of other pollutants in co-environmental media [53]. Long-term exposure to high concentrations of MPs can significantly diminish plant survival and reproductive capacities, threatening the stability and biodiversity of wetland ecosystems [47]. Increasing evidence indicates that MP accumulation in the rhizosphere can either reduce or enhance root respiration rates and exudate consumption rates, contingent upon MP type and exudate compounds, playing a crucial role in reconstructing the rhizosphere microbial community composition [48]. This is because MP-induced alterations in root exudate amino acids can serve as a dominant nitrogen nutrient source for microbial metabolism [54]. In summary, different types of MPs exhibit distinct mechanisms of influence on microbial community structure and plant root function, attributed to variations in degradability or the toxicity of released substances. However, most current research focuses on short-term pot experiments, which markedly differ from the complex interactions among multiple MPs and co-existent pollutants in long-term field studies.

## 4. Microplastic Impacts on Carbon and Nutrient Cycling

### 4.1. Organic Carbon Behavior Under Microplastic Exposure

The accumulation of MPs in soils has been shown to generally increase organic matter and total organic carbon by an average of approximately 30% and 35%, respectively, potentially enhancing the abundance of carbon-degradation-related genes and promoting carbon release [31]. Conversely, significant oxidative stress and photosynthesis inhibition induced by MPs often result in more than a 30% decrease in plant biomass, thereby reducing carbon sequestration during plant growth [31,55]. Furthermore, MP exposure can inhibit plant growth and nutrient cycling due to altered microbial community composition in wetland sediments, leading to decreased nitrogen and carbon content in aquatic plant leaves [24]. The multifaceted impacts of MPs on organic carbon components within wetland ecosystems weaken carbon sequestration capacity, disrupt nutrient cycling, and pose risks to ecosystem health and climate change mitigation [22,56]. Specifically, MPs can directly increase sedimentary carbon stocks through their absorption by vegetation and organisms in wetland sediments. However, this increase may alter carbon cycle dynamics, affecting both carbon fixation and emission processes [57]. In particular, small-sized MPs (<1 mm) found in wetlands dominated by seagrass beds and mangroves mimic the behavior of capturing external blue carbon sources, such as drifting algae, plant debris, and terrestrial organic matter [58].

Nitrogen limitation, resulting from MP-induced alterations in microbial communities, slows down plant growth, impacting carbon sequestration efficiency and altering the carbon-to-nitrogen ratio in wetland ecosystems [59]. Moreover, MP exposure exacerbates nitrogen retention, indirectly influencing organic carbon decomposition processes [59,60]. Both conventional and biodegradable MPs facilitate anaerobic processes in sediments, with biodegradable MPs serving as a significant carbon source for microbial communities, accelerating nutrient turnover and loss [61,62]. The degradation of MPs promotes greenhouse gas emissions, especially smaller MP particles, which accelerate CH_4_ and CO_2_ release [63]. Interestingly, Chen et al. [64] reported that MPs increase DOM humification, promoting high-molecular-weight components and suppressing DOM bioavailability, thereby indirectly reducing CO_2_ emissions. Hitherto, significant challenges remain in studying the release processes and influencing factors of MPs-DOM in different wetland types. The aging process of MPs in natural environments and their ecological risks should not be ignored due to the release of significant amounts of MPs-DOM. This new carbon source, rich in functional groups like carboxyl and hydroxyl groups, can directly or indirectly affect the fate and transformation of iron minerals and other compounds, potentially influencing microbial activity and carbon cycling [65,66].

### 4.2. Specific Roles of Microplastic-Derived Dissolved Organic Matter

In natural environments, plastics undergo various oxidation reactions, including illumination, ultraviolet radiation, and mineral–microbial interactions, leading to the release of dissolved organic matter (DOM) due to the breakdown of MP-containing organic compounds (e.g., additives and major polymers), which accounts for approximately 70% of plastic fragments by mass [67]. It has been reported that the annual release of MPs-DOM may constitute about 10% of the total organic matter content on the surface of seawater [68]. The promotion of photodegradation and alterations in microbial metabolic activity are considered pivotal factors controlling microbial transformation processes and DOM evolution in soil and sediment environments. These processes collectively influence carbon fixation, decomposition, and storage [64,69]. Previous studies have highlighted a feedback loop between MPs-DOM release and microbial metabolic activity, where enrichment with MPs promotes the degradation of low-aromatic active components, thereby enhancing aromaticity and oxidation levels [70]. Biodegradable MPs, owing to their oxygen-rich molecular structures, are particularly effective at releasing DOM and oxygenated structures through hydrolysis [71]. Upon entering soil environments, it is primarily bacteria rather than fungi that metabolize MPs-DOM, indicating that specific members of the soil microbiome can utilize MPs-DOM [72]. This preference is likely because bacteria play key roles in rapid carbon cycling pathways in soils, whereas fungi predominantly break down recalcitrant organic matter [70,73].

Short-term exposure to MPs reduces the bioavailability of DOM in sediments, inhibiting CO_2_ emissions; however, this effect diminishes over time as sediment microbes begin to utilize aging MPs as a fresh carbon source, promoting organic carbon mineralization [64]. Furthermore, while MP exposure may reduce carbon emissions from sediments, it also decreases DOM bioavailability, which can lead to reduced carbon biodegradation and promote organic phosphorus mineralization by microorganisms, potentially causing eutrophication risks [74]. When MPs and benthic organisms in wetland sediments synergistically affect the geochemical characteristics of DOM, they create unique habitats or ecological niches, altering the microbial community composition in sediments [75,76]. Moreover, MPs influence key microorganisms in sediments through the leaching of harmful substances and biological disturbances, particularly favoring microorganisms that are tolerant of MPs, which may possess the ability to degrade MPs [18]. These microorganisms can utilize carbon from MPs to generate biomass and promote the formation of degradation products, affecting the DOM characteristics of the sediment and further impacting carbon metabolism pathways and the cycling of other elements such as nitrogen and phosphorus [77]. Additionally, photodegradation reactions significantly impact the release of MPs-DOM, especially from biodegradable MPs, enhancing photochemical reactivity. Chromophores generated during the aging process (e.g., oxygenated functional groups) increase light absorption and ROS production. This photochemical reaction not only affects the aging process of MPs but also influences the properties of DOM, indirectly affecting microbial metabolic activity and carbon cycling pathways [78,79]. Although there is increasing attention from researchers on the negative impacts of MPs-DOM on the microbial carbon cycle in waters, soils, and sediments, many aspects remain unclear, particularly regarding different vegetation types and water–soil interface environments within wetland ecosystems.

## 5. Adverse Impacts of Microplastics on Wetland Carbon Sequestration

While studies have indicated that MPs can augment the carbon content in coastal wetland sediments, acting as a novel carbon storage medium, this enhancement presents both opportunities and challenges [20]. In the short term, MPs may boost carbon sink capacity; however, they alter sediment structure, potentially compromising long-term carbon stability. Moreover, MPs disrupt symbiotic relationships between plants and microorganisms, diminishing plants’ carbon fixation capabilities due to a reduction in beneficial bacteria and an increase in pathogenic microorganisms within soils or sediments [51].

### 5.1. Disruption of Microbial Carbon Metabolism

The accumulation of MPs in waters or sediments forms biofilms with various microbial community colonizations depending on MP types and original carbon sources in the environments. These microbial communities are enriched with plastic-degrading bacteria or related carbon/nitrogen metabolism functional genes [80]. Similarly, the introduction of MPs into wetland sediments can alter microbial metabolism through increased surface area for microbial attachment, thereby enhancing enzyme active sites [53]. These changes can lead to disruptions in nutrient utilization and carbon assimilation, possibly degrading essential wetland services. The selective colonization of certain archaea, including methanogens, on MP surfaces modifies the microbial community structure, influencing organic carbon decomposition and CH_4_ generation [81]. Furthermore, MPs may serve as new habitats for methanogens or alter their metabolic activities by changing sediment pH and other chemical conditions, thus affecting CH_4_ production and emissions from wetlands [7]. Yang et al. [82] reported that MPs also impact key microbial groups involved in nitrogen cycling, like ammonia-oxidizing bacteria and denitrifying bacteria, altering N_2_O production and overall carbon cycling dynamics. Additionally, chemicals adsorbed onto or released from MPs stimulate specific microbial growth, accelerating organic carbon degradation and increasing CO_2_ release risks [23]. Researchers have identified several kinds of microbial communities, such as *Firmicutes*, *Proteobacteria*, and *Actinobacteria*, capable of degrading plastics from various environments. Notably, some bacterial species (e.g., *Pseudomonas*, *Escherichia*, and *Bacillus*) are expected to degrade recalcitrant polymers [83,84]. During the biodegradation processes for plastics, produced microbial extracellular enzymes or free radical catalysis can further decompose plastics into smaller molecules or even monomer compounds, which can eventually be mineralized into CO_2_ under aerobic conditions or CH_4_ under anaerobic conditions [85]. Nonetheless, further investigation into these specific microorganisms and their metabolic activities is needed to explore MP degradation and pollution remediation technologies in various wetland ecosystems.

### 5.2. Amplification of Greenhouse Gas Emissions

Microplastic-induced alterations in microbial metabolic activities and carbon use efficiency significantly influence greenhouse gas emissions, particularly in the anaerobic environments prevalent in wetlands. Despite minimal impacts on microbial biomass, MPs notably affect soil microbial biomass carbon and the metabolic quotient (qCO_2_) more in anaerobic environments than in aerobic surface soils [60]. Polylactic acid (PLA) MP exposure increases CO_2_ emissions from deep soil due to its heteroatom-rich composition, facilitating hydrolysis and enzymatic reactions and making it easier for microorganisms to metabolize and ultimately mineralize into CO_2_ [86]. Biodegradable MPs exacerbate N_2_O and CH_4_ production in urban lake sediments compared to traditional non-biodegradable MPs, primarily due to enhanced denitrification processes stimulated by biodegradable MPs [87]. These MPs create oxygen-limited micro-environments with increased dissolved organic carbon, promoting heterotrophic denitrification and N_2_O production via the nitrification–denitrification pathway [88]. Additionally, biodegradable MPs increase CH_4_ production by providing additional organic carbon substrates, stimulating microbial CH_4_ metabolism [87,89]. The accumulation of MPs in coastal wetlands attracts diverse microbial groups, modifying microbial community structures during litter decomposition and increasing proportions of methanogens and denitrifying bacteria, directly or indirectly boosting greenhouse gas production [21,90]. Notably, conventional and biodegradable MPs promote CH_4_ emissions through distinct mechanisms: conventional MPs inhibit CH_4_ consumption without significantly affecting production, whereas biodegradable MPs accelerate methanogen activity and inhibit CH_4_ oxidation, having a greater effect on CH_4_ emissions [21]. As biodegradable plastics increasingly replace nondegradable counterparts, future research should focus on their impacts on microbial communities and metabolic functions, especially concerning long-term ecological effects on greenhouse gas emissions.

## 6. Mitigating Microplastic Pollution to Protect Wetland Ecosystems

The introduction of MPs into blue carbon ecosystems, such as seagrass beds and mangroves, poses significant threats to their health, functionality, and even global climate change mitigation efforts [58]. Therefore, mitigating MP pollution in wetlands has become a critical task aimed at preserving these vital resources and restoring their carbon sequestration capabilities [91,92]. Utilizing microbial degradation and ecological engineering methods to mitigate the impact of MPs on wetland functions, microbial community structures, and carbon cycling is considered a promising strategy [93,94]. Auta et al. [95] isolated *Bacillus* strains from mangrove environments that demonstrated significant degradation capabilities for polyethylene and polypropylene, proving their adaptability to complex wetland conditions. Further research by the same group explored the growth dynamics and biocorrosion effects of *Bacillus* and *Rhodococcus* on polypropylene MPs in mangrove sediments, showing rapid proliferation under suitable conditions and accelerated MP aging through enzymatic activity [96]. Given earthworms’ role in soil ecosystems, Lwanga et al. [97] confirmed that bacteria extracted from earthworm intestines could degrade low-density polyethylene, suggesting that introducing earthworms or other soil organisms may enhance natural MP degradation in wetlands.

Furthermore, combining physical filtration, chemical treatment, and bioremediation strategies is essential for mitigating the interactions between MPs and other pollutants, as well as their cumulative impacts on wetland ecosystems [98]. Coastal zones, identified as major sinks for MPs, offer unique opportunities for effective pollution control using the biomass richness and diversity of tidal vegetation. These plants can intercept MP particles at the land–sea interface over large spatial and temporal scales, especially in tropical and subtropical mangrove ecosystems with specialized root structures [99]. However, further investigation is required to understand the adsorption capacities of different aquatic plants in estuaries and coastal wetlands, along with the interactions between rhizosphere microorganisms and MP degradation. Through such efforts, we can better protect wetland ecosystems and enhance their resilience against the adverse impacts of MPs, ensuring their continued contribution to global carbon sequestration and biodiversity conservation.

## 7. Conclusions and Perspectives

Wetland ecosystems, serving as critical ecological services and carbon sinks, are facing unprecedented challenges from MP pollution. The accumulation of MPs in wetlands disrupts soil structures, microbial communities, and carbon metabolism processes, interfering with nutrient cycles, inhibiting plant biomass, and promoting greenhouse gas emissions. Microplastics provide additional habitats for various microbial groups, including methanogens and denitrifying bacteria, indirectly enhancing CH_4_ and N_2_O production. Long-term exposure to MPs may damage plant roots, impair photosynthesis, and reduce carbon sequestration efficiency. While some studies suggest that MPs might temporarily increase carbon sink potential, this effect is unsustainable due to the eventual mineralization of organic carbon and release of CO_2_. More importantly, MPs-DOM exerts significant potential for disturbing the organic carbon pool by accelerating mineralization, thereby enhancing CO_2_ or CH_4_ from the waters or sediments of wetlands. Additionally, due to the different additives or co-existent pollutants within MPs, the interactions of MPs-DOM with organisms and the consequent biological toxicity are varied and complex. Future research should focus on understanding the specific mechanisms by which different types of MPs influence microbial metabolic activities and carbon sequestration in diverse wetland environments. The key recommendations include the following:

(1) The distinguished contents of MPs in wetlands reported across different studies demonstrate a large unknown in the traceability of MPs and their by-products. Future research should pay attention to the standardization of analysis and detection methods for MPs and corresponding by-products, uncovering their distribution in multiple environments such as waters, sediments, soil–plant systems, and water–soil interfaces in wetlands, while exploring the physical, chemical, and biological factors influencing the migration of MPs and the formation of by-products.
(2) Although studies have analyzed the variation in the interception ability of different plant species for MPs in wetlands, the translocation of these MPs in different plants remains unclear, hindering the analysis of MPs sinks in plant systems and their effects on plant growth. This also impedes accurately assessing the impacts of MPs on other organisms and human health through the food chain.
(3) The toxicity of additives and laden substances released from MPs during decomposition varies based on the types, particle sizes, and shapes of MPs, posing various influences on microbial habitats, biofilm formation, and plant rhizosphere development. Future research should focus more on the impacts of different MP types (e.g., polyethylene, polypropylene, biodegradable plastics) on microbial metabolic activity under natural wetland conditions, considering their implications for long-term carbon sequestration and global climate change.
(4) Examining the direct effects of MPs-DOM released from both pristine and aged MPs on microbial community structure, as well as the indirect effects on microbial metabolism by affecting the geochemical behavior of organic matter and physicochemical properties in soils/sediments, is more noteworthy than the adverse effects of MPs themselves, especially for biodegradable MPs.
(5) Isotope-labeled experiments and the metagenomic tracking of element transformation during sediment metabolism can be employed in future investigations, potentially providing valuable insights into the mechanisms of additives or DOM components released from MPs and their effects on MP degradation and carbon cycling.
(6) Identifying microbial species and developing functional microbial agents capable of degrading MPs, along with assessing their environmental adaptation and service life, represent great challenges but are essential for constructing tailored ecological engineering techniques for field remediation.

Overall, this review synthesizes the current findings on MPs’ impact on wetland carbon cycling, offering valuable insights for future MP pollution control and prevention research. It underscores the importance of protecting wetland ecosystems’ carbon sequestration capacities and promoting sustainable development amidst the global challenges posed by MP pollution.

## Data Availability

No new data were created or analyzed in this study. Data sharing is not applicable to this article.
